# Deriving monetary value of quality-adjusted life years through life extension from the value of a statistical life

**DOI:** 10.1038/s41598-025-29794-6

**Published:** 2025-12-01

**Authors:** Yusuke Tanizawa, Kazuya Ito, Ryuta Takashima

**Affiliations:** Department of Industrial and Systems Engineering, Faculty of Science and Technology, Tokyo University of Science, 2641 Yamazaki, Noda, Chiba 278-8510 Japan

**Keywords:** Quality-adjusted life years, Value of statistical life, Healthcare, Cost-benefit analysis, Health care economics, Health policy, Public health, Quality of life

## Abstract

To address the recent rise in healthcare expenditure due to an aging population, the rational allocation and efficient use of resources, based on scientific evidence, have become indispensable. This study proposes an alternative framework for estimating quality-adjusted life years (QALY) based on the value of statistical life, which can be used for cost-benefit analysis (CBA) of policy interventions and the efficient allocation of healthcare resources. Specifically, we estimate the monetary value of a QALY based solely on life extension. We assess the accuracy of conventional QALY estimates while proposing a new, more rational, and flexible QALY estimation that combines age and scenario factors. Our numerical analysis suggests that updating QALY by considering regional characteristics such as population, age distribution, and changes in quality of life (QoL) can lead to a more accurate CBA. This metric provides information for decision-making in policy budgeting based on scientific evidence and suggests that this approach may contribute to a more efficient allocation of healthcare resources. Furthermore, increasing the proportion of healthy individuals with a gradual decline in QoL may support efforts to reduce healthcare expenditure.

## Introduction

In recent years, advances in medical technology and improvements in public health have led to an increase in aging populations in many countries^1,2^. This demographic shift poses the challenge of increasing social costs, particularly healthcare expenses. For instance, Japan, the focus of this study, has one of the highest life expectancies and healthy life expectancies in the world, while simultaneously experiencing a significant decline in birth rates. By 2040, Japan’s healthcare expenses are expected to be more than 1.8 times those of 2018.^3^ There is a growing concern that current trends could render healthcare systems unsustainable, prompting discussions on institutional reforms and policy support globally^4–8^. In the coming decades, many countries, particularly those in the Organization for Economic Co-operation and Development (OECD), are expected to follow similar trajectories of population aging and low birth rates. In this context, the imperative to incorporate healthy life expectancy into policy decision-making will only grow stronger^9–11^. Therefore, building a sustainable healthcare system for the future requires the prudent allocation and effective use of resources based on scientific evidence^12–16^. In our study, the term scientific evidence refers to systematically generated research findings that are used to inform healthcare decisions, in accordance with the principles of evidence-based healthcare and health policy^17–19^.

In the healthcare sector, cost-benefit analysis (CBA) using measures such as the value of statistical life (VSL) and quality-adjusted life years (QALY) is commonly employed to guide policy interventions and the efficient allocation of healthcare resources^20–24^. The VSL is calculated based on willingness to pay for mortality risk reduction and is widely used in CBA to evaluate the economic benefits of a policy^25^. The QALY, which considers both quality of life (QoL) and life expectancy, equates one QALY to 1 year of life in perfect health (QoL = 1)^26,27^. The VSL and QALY are considered to be closely related, and research on their relationship has been active in recent years^28^. This measure allows for cross-sectional comparisons of different healthcare policies and is widely used in many countries as a standard metric for public health policies and resource allocation decisions^29–31^. By employing QALY-based CBA, policymakers can quantitatively assess the effectiveness of healthcare interventions based on scientific evidence, thereby facilitating informed decision-making^26,32–36^. For instance, the UK’s National Institute for Health and Care Excellence (NICE) uses QALY to assess pharmaceutical and medical technologies, providing guidelines for the effective use of limited healthcare resources^37^.

However, the QALY has several limitations. For example, it applies uniformly across different age groups, despite significant differences in health status and life expectancy between younger and older individuals^23,38–40^. The current QALY-based CBA may not adequately account for age-specific differences, potentially leading to biased results. Additionally, QALY values are often derived based on practices from other countries without fully considering regional characteristics such as population, economic conditions, and age distribution. The lack of consistency and insufficient bases in the setting of QALY has been highlighted in many studies^41–44^. These issues undermine the accuracy of QALY-based CBA and policy evaluations, potentially leading to inefficient allocation of healthcare resources and suboptimal social expenditure^4,34,45^.

To build a sustainable healthcare system, it is essential to derive robust QALY estimates that will enable policymakers to make scientifically grounded decisions. This study aims to present a QALY metric that considers age-specific health status (QoL) and life expectancy by deriving QALY from VSL. We model the VSL-based QALY and demonstrate its effectiveness through a scenario and policy evaluation analysis. In this study, we focus our analysis on the monetary value of a QALY that arises solely from life extension without incorporating QoL improvements and present the results of VSL, QALY, and policy cost reduction, using socioeconomic data from Japan. While our analysis remains within the positive scope in terms of methodology, focusing on quantifying the monetary value of a QALY for healthcare policy evaluation, it also offers normative implications by informing the effective allocation of healthcare resources.

## Results

We begin by presenting the results of VSL (VSL-years) for each scenario in our modeling framework. We then present the age-specific monetary value of a QALY and its population-weighted averages under each scenario. By comparing the monetary value of a QALY across scenarios, we characterize the age-dependent range of QALY. Finally, we present an example of policy evaluation focused on cost reductions. Throughout, our exposition is linked to the Methods section.

### Estimates of VSL-year and VSL by scenario

**Fig. 1:**
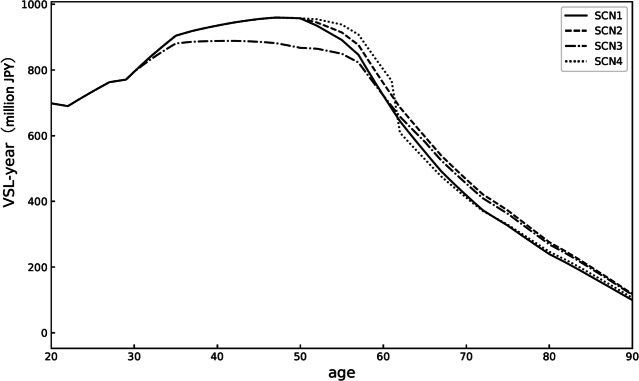
VSL-year (million JPY) for each age group under various scenarios in Japan, calculated from socioeconomic data on wages, consumption, survival rates, and population. SCN1, SCN2, SCN3, and SCN4 are depicted by solid, dashed, dash-dot, and dotted lines, respectively.

Figure 1 illustrates the VSL-year for each age group under various scenarios. According to these results, the VSL for each scenario is as follows: SCN1: 457.6 million JPY, SCN2: 468.6 million JPY, SCN3: 452.9 million JPY, and SCN4: 462.8 million JPY. These results indicate that the VSL ranks in the order of SCN2, SCN4, SCN1, and SCN3.

### Estimates of VSL-based QALY

We calculate the value of one QALY for representative ages across different scenarios based on VSL, as shown in Table 1. The population-weighted average is derived by multiplying the QALY values for each age group by the respective population distribution and then averaging them. Across all scenarios, the QALY increases with age. When comparing the scenarios, the estimated QALY values decrease in the order of SCN1, SCN4, SCN3, and SCN2.

**Table 1:** The value of VSL-based QALY (million JPY) for representative ages, and population-weighted averages using Japan’s population distribution in each scenario.

Age	SCN1	SCN2	SCN3	SCN4
20	3.54	3.11	3.74	3.59
30	3.90	3.42	4.12	3.96
40	4.27	3.73	5.00	4.34
50	4.61	4.01	5.99	4.68
60	5.91	4.56	6.34	4.88
70	7.47	4.98	6.90	7.96
80	9.43	4.94	7.78	9.11
Population-weighted average	6.05	4.08	4.66	5.99

**Fig. 2:**
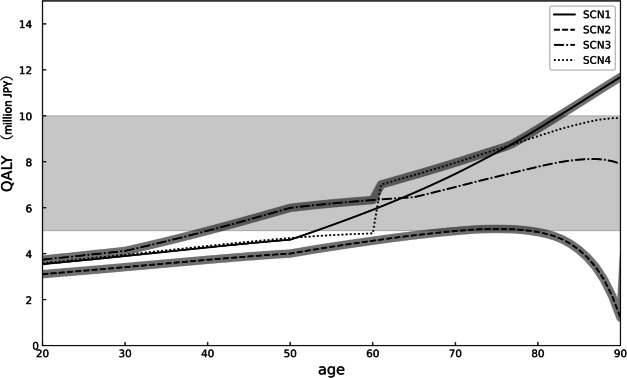
The value of QALY (million JPY) comparison across age groups and scenarios highlighting highest and lowest values. SCN1, SCN2, SCN3, and SCN4 are depicted by solid, dashed, dash-dot, and dotted lines, respectively. The gray shaded area represents the range of typical QALY estimates in Japan. The two gray lines indicate the minimum and maximum scenario for QALY estimates at each age.

Figure 2 presents the value of QALY for each age group under different scenarios. The shaded area represents the range of QALY values commonly used today, whereas the thick line traces the scenarios with the highest and lowest QALY values for each age group. From ages 20 to 60, SCN3 shows the highest QALY values, followed by SCN4 and SCN1. Conversely, SCN2 consistently exhibits the lowest values.

### Evaluation of cost reductions by comparing conventional QALYs to those of this study

Table 2 shows the cost reduction obtained using the difference between conventional QALY and the QALY estimated in this study in various scenarios. When the QALY is uniformly estimated at 5 million JPY, a higher proportion of SCN1 results in a negative cost reduction, whereas an increased proportion of SCN2 results in a positive cost reduction.

**Table 2:** Comparison of policy cost reduction effects between conventional and this study’s QALYs under various scenario proportions.

Case	Proportion (%)				Cost reduction (trillion JPY)
	SCN1	SCN2	SCN3	SCN4	
1	100	0	0	0	− 107.8
2	70	0	15	15	− 105.4
3	50	20	15	15	− 65.1
4	30	40	15	15	− 24.6
5	10	60	15	15	15.8

Proportion indicates the percentage of the population that belongs to each scenario. Cost reduction means excess when positive and insufficiency when negative.

## Discussion

The VSL calculated in this study falls within the range of typical values across all scenarios, and the general shape of the VSL-year curve also peaks between the ages of 30 and 50, which is consistent with previous research^46–48^. Comparing the scenarios shows that a longer period of healthy living, indicated by a higher QoL over a longer duration, increased the VSL. Specifically, in SCN2, which assumes a 1% reduction in health decline after the age of 50, the VSL-year (utility) at each age is generally higher, resulting in a larger total VSL. Comparing SCN1 and SCN2, the difference in this value is more than 10 million JPY over a lifetime. Conversely, in SCN3, where QoL declines from a relatively young age, the VSL-year values for individuals in their 40s and 50s are lower, leading to a reduced overall VSL. These results suggest that in regions with a high prevalence of SCN3 conditions, concentrating medical resources on these preventive measures could be particularly effective in regions with a high prevalence of SCNs.

The current value used for CBA in Japan is 5 million JPY per QALY, mainly based on figures from other countries^30,49,50^. The population-weighted average QALY in this study (Eqs. (2) and (3)) approximates this value across all scenarios. The alignment in the shape and estimates of the VSL with those in previous studies for each scenario supports the use of conventional QALY in CBA. For example, if we assume the proportions of each scenario to be 50% for SCN1, 20% for SCN2, 15% for SCN3, and 15% for SCN4, the estimate for 1 QALY in Japan would be calculated as follows: $6.05 \times 0.5 + 4.08 \times 0.2 + 4.66 \times 0.15 + 5.99\times 0.15 = 5.44$ million JPY. However, similar to previous research, the monetary value of a QALY varies by age, with higher values observed at older ages^51–53^. Since the future value becomes smaller than the present one due to discounting, the old have a large impact on a life expectancy gain of 1 year compared to the young. This implies that the old recognize the value of the additional life expectancy earlier than the young. Thus, the benefit from a life expectancy gain increases with age. Additionally, we can address how to determine QALY for each age group by considering various QoL scenarios. First, when considering the scenario with the highest value at each age, the upper bound for QALY is SCN3 from ages 20 to 60, SCN4 from ages 60 to 75, and SCN1 thereafter. This pattern indicates that QALY values increase initially with SCN3 due to gradual health decline from a young age, followed by SCN4 with a sharp QoL decline around the 60 s, and SCN1 with an increase in the value of one QALY in older age groups. In contrast, a lower bound is observed for SCN2. In this scenario, QoL does not decline significantly even in old age because health is maintained throughout life, resulting in a smaller effect when obtaining an additional QALY than in the other scenarios. These upper and lower bounds represent extreme cases in which the entire population falls into a single scenario at each age. However, in reality, the population is distributed across various scenarios in certain proportions. Hence, we suggest that, regardless of the weights (proportion) of each scenario, an estimate of the monetary value of QALY is likely to fall within the upper and lower bounds.

We also suggest that the use of an age-adjusted QALY could enable a more reasonable and efficient CBA. For instance, a healthcare policy with an estimated cost of 6 million JPY per QALY would result in negative cost-effectiveness if a uniform value of 5 million JPY per QALY is applied across all ages, meaning that the policy would not be implemented. However, our study shows that cost-effectiveness varies by age, which influences decision-making regarding policy implementation. For example, a policy with an estimated cost of 6 million JPY targeting individuals in their 20–30 s would be ineffective, as the value of increasing one QALY is in the range of 3–4 million JPY. Conversely, the same policy targeting individuals in their 70 s and 80 s could be effective, except for SCN2. This indicates that cost-effectiveness can be positive or negative depending on the target age group, emphasizing the need for accurate CBA. Policies targeting younger individuals should result in lower QALY, whereas those targeting older individuals should result in higher QALY.

A more advanced discussion involves the extension of a healthy life expectancy. We demonstrate that extending healthy life expectancy can lower QALYs and in turn, reduce policy costs from the results of Table 2. This can be easily understood by considering the extreme case in which the entire population shifts from SCN1 to SCN2: The value of a QALY decreases from 6.05 to 4.08 million JPY with an extended healthy life expectancy, thereby reducing the budget needed to increase one QALY. In other words, the value of a population-weighted average QALY decreases from 6.05 to 4.08 million JPY with an increase in the number of healthy individuals experiencing a gradual decline in QoL, thus requiring a smaller budget to increase one QALY. Put differently, while the benchmark for cost-effective analysis in healthcare policy was previously set at 6.05 million JPY to obtain benefits, it now shifts to 4.08 million JPY. This shift implies that policy decisions to increase a QALY are made at a lower threshold, meaning that a QALY can be increased at a lower cost. Consequently, an increase in the number of healthy individuals with a slower decline in QoL, that is, an extension of healthy life expectancy, results in reduced social costs. However, this study does not explicitly incorporate the potential costs of preventive programs, education, or social policies. Including such costs would allow for a more comprehensive assessment.

The cost reduction results illustrate the impact of accurately estimating QALY for Japan’s entire population under various scenario proportions based on the difference between our QALY and the conventional QALY. Case 1 assumes that the entire population follows SCN1 for lifetime changes in QoL, and is presented as an example in a previous study^51,54^. This case shows negative cost reduction, indicating that the current QALY estimate is too low and would require an additional budget of 107.8 trillion JPY for implementing policies to increase QALY by one unit across Japan. Cases 2 through 5 assume that policy measures increase the proportion of SCN2, that is, healthy life expectancy, under a certain number of SCN3 and SCN4. When the proportion of SCN2 is low, the cost reduction remains negative. However, in Case 5, in which the proportion of SCN2 increases significantly, the cost reduction turns positive. These results suggest that if the number of people with an extended healthy life expectancy and a more gradual decline in QoL increases, the current QALY estimate becomes excessive, thereby reducing the budget required for healthcare policies. In other words, the lower cost to increase one QALY reduces the social costs allocated to healthcare resources (or the budget to increase one QALY across Japan). Therefore, when a larger proportion of the population maintains good health throughout their lives, CBA for policy implementation should be conducted at lower values. We suggest that more realistic QALY estimates can be achieved by appropriately weighting QoL scenario proportions.

The VSL-based QALY method proposed in this study can be applied to any region by utilizing socioeconomic data specific to the target area. It can also be adapted for longitudinal and historical analysis. This allows for the comparison of QALY across regions and the observation of temporal changes. However, the VSL measurement in this study cannot fully capture the impact of specific risks, such as cancer, as well as other attributes like income, education, and family structure.

### Limitation and future work

Our method for estimating QALY warrants further investigation to test its validity when applied to actual policy evaluations. Especially, our future study might include the issue of double counting^55–57^. Our current formulation implicitly accounts for the utility of consumption of both LEV and QoL, which potentially leads to double counting^58,59^. A detailed evaluation of consumption-related double counting would represent independent research. Accordingly, we have identified it here as an important topic for future work.

Further examination of the monetary value of QALY estimates using alternative functional forms would be valuable. In particular, future work could examine modeling utility as a nonlinear function of health, incorporating interactions between income and health, and employing alternative discounting approaches.

Finally, QALY estimates need to be justified using socioeconomic data from a broader range of countries and regions. Future research may also need to consider additional factors beyond those we have already included, such as cultural influences, social values, and medical advancements. To achieve this, the application and refinement of country- or region-specific scenarios could be an important extension. Scenarios involving improvements in QoL could be considered in future studies. One such scenario might include enhanced physical and mental health resulting from the development of new healthcare policies or medications.

## Conclusion

Recent advances in medical technology and improvements in public health have led to people living longer and, consequently, increased healthcare expenditure as populations age. If this trend continues, the current healthcare system may become unsustainable. Therefore, the rational allocation and effective use of resources based on scientific evidence using QALYs are indispensable. Given the anticipation that life expectancies will continue to increase globally, an analysis focused on Japan could provide valuable insights for the development of future policies and indicators globally.

Our proposed VSL-based QALY estimation method contributes to the interpretability and policy applicability by incorporating demographic elements such as population distribution, age groups, and QoL. We identify three key findings in this study. First, the conventional QALY estimate exhibits a certain level of consistency compared to our proposed QALY. Second, we demonstrate the variations and ranges in QALY estimates based on age and QoL, indicating that these findings contribute to a more rational CBA for the efficient use of health expenditure. Finally, from a cost-reduction perspective, we illustrate the differences between conventional QALY and our proposed QALY, emphasizing the potential for extending healthy life expectancy to reduce healthcare expenditure. This metric provides one tool for rational decision-making for policy budgeting based on scientific evidence, highlighting the potential for greater efficiency in healthcare resource allocation. In a future world of aging populations and declining birth rates, the intrinsic value of QALY is likely to increase over time. The policy evaluation example presented here for Japan serves as a model case for deriving the monetary value of a QALY under such a situation, offering valuable insights into future global demographic shifts.

## Methods

In this section, we first define the economic model of the VSL and use it to derive the value of life extension (LEV). We then combine LEV with QoL measures to calculate the monetary value of a QALY for any given age and scenario. After detailing our QoL scenario setting, we proceed to describe the methodology for conducting policy evaluations using the QALY.

### Economic model of VSL

In this study, we estimate the value of the QALY by considering QoL and discuss the potential for policy evaluation using these estimates. The economic model of VSL is based on previous research and incorporates socioeconomic data and weighs the utility derived from consumption and income by applying QoL to reflect health status^51,54^. Furthermore, it calculates VSL by converting the expected utility based on survival rates into present values in continuous time. This model allows for the representation of lifetime utility as a smooth curve. The VSL at any given age *a* in scenario *m* can be calculated as follows:

1 $$\begin{aligned} VSL_{m}(a)=\int _{a}^{\infty } e^{-r(t-a)}\left( y^{F}(t)+\Phi _{m}(z(t))c^{F}(t)\right) S(t,a)dt \end{aligned}$$

where *e* is the base of the natural logarithm. $c^{F}(t)$ represents full consumption, $y^{F}(t)$ represents full income, and *r* denotes the interest rate. The primary assumption in our model framework is the interest rate, which we set at 1% based on previous research^54^. The results of the sensitivity analysis regarding the interest rate are shown in Supplementary Table S1. $\Phi _{m}(z_{m}(t))$ is the elasticity of substitution and is used in this model as a parameter to replace the utility of consumption and leisure with that of income and consumption, considering health status. We define $\Phi _m(z(t)) = \frac{u(z(t))}{z(t)u'(z(t))}-1$, where *z*(*t*) denotes the composite good that combines consumption and leisure at age *t*, and *u*(*z*(*t*)) is the utility derived from this composite good. Our calibration of $\Phi _m(z(t))$ follows Murphy and Topel^54^, while we introduce QoL scenarios that modify the trajectory of *z*(*t*) and lead to scenario-specific values of $\Phi _m(z(t))$. Additionally, *S*(*t*, *a*) is a function of the survival rate from age *a* to *t*. These elements allow the model to reflect variations in lifetime utility considering health status. The VSL at any given age is expressed as the VSL year, and the sum of all VSL years corresponds to the total VSL^60^.

### Derivation of VSL-based QALY

We derive a new metric, the VSL-based QALY, for enhanced healthcare CBA. Using the economic model of VSL, the value of a QALY can be calculated for any given age. In other words, the VSL and QALY have a one-to-one correspondence. The LEV is defined as an increase in the unit area of the survival rate function at the age at which the extension occurs. At any given age, by dividing the LEV by the QoL, we can determine the LEV in a state with a QoL of 1, that is, the value of a QALY. The LEV at age *a* in scenario *m* can be expressed as:

2 $$\begin{aligned} LEV_{m}(a)=\int _{a}^{\infty } e^{-r(t-a)}\left( y^{F}(t)+\Phi _{m}(z(t))c^{F}(t)\right) (S(t-1,a)-S(t,a))dt \end{aligned}$$

The monetary value of a QALY at age *a* can be expressed using the Eq. (2) as follows:

3 $$\begin{aligned} QALY_{m}(a)=\frac{LEV_{m}(a)}{H_{m}(a)} \end{aligned}$$

$H_{m}(a)$ is a function that represents QoL, defined between 0 and 1. A value closer to 1 indicates a healthier state, whereas a value of 0 indicates death. At age *a*, the LEV by 1 year can be calculated by dividing it by the QoL, which represents the value of extending a state with a QoL of 1 for 1 year, that is, the value of one QALY. This enables consistent comparisons of the value of one QALY across different ages. Note that both the numerator and denominator of Eq. (3) involve the utility of consumption, which may raise concerns due to the potential for double counting^58^. An important related study to our research is Hammitt^28^. Although the mathematical structures of Hammitt’s value per quality-adjusted life year (VQALY) and our value of QALY differ, they are essentially equivalent and share the same interpretation (see Supplementary Note). Additionally, we examined the potential impact of alternative functional forms on our estimates of VSL, LEV, and the monetary value of a QALY. Specifically, we recalculated the monetary value of a QALY under three alternative assumptions. First, we adopted a logarithmic utility function, recalculating $\Phi _m(z(t))$ with $u(z(t))=\ln (z(t))$ (Supplementary Table S2). Second, we employed a constant relative risk aversion (CRRA) utility function to reflect risk-averse preferences, recalculating $\Phi _m(z(t))$ with $u(z(t))=(z(t)^{1-\eta }-1)/(1-\eta )$ with $\eta$=0.5 (Supplementary Table S3). Third, we applied hyperbolic discounting by replacing the discount factor $e^{-r(t-a)}$ with $1/(1+\kappa (t-a))$, setting $\kappa =0.01$ for consistency with the baseline interest rate *r* (Supplementary Table S4). In all of these additional analyses, the monetary value of a QALY did not differ substantially from our main results, and the relative ordering across scenarios remained unchanged.

### QoL scenario setting

We set several scenarios to calculate a more realistic QALY. These scenarios illustrate that the trajectory of an individual’s QoL over their lifetime is not uniform and can reflect changes in QoL due to various diseases and other factors. By examining these scenarios, we can obtain a more flexible QALY calculation and accommodate diverse health conditions and lifestyles, while clarifying the meaning of QALY.

**Fig. 3:**
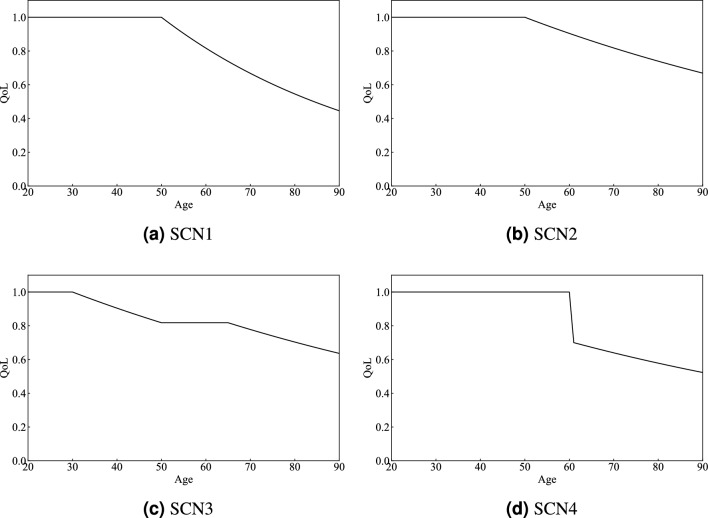
Various scenarios of QoL trajectories for each age group, considering factors of health decline. SCN1: QoL decreases by 2% annually after age 50. SCN2: QoL decreases by 1% annually after age 50. SCN3: QoL decreases by 1% annually starting at age 30, remains constant between ages 50 and 70, and then decreases by 1% annually after age 70. SCN4: QoL drops sharply at age 60 and then decreases by 1% annually thereafter..

The scenarios established were as follows:

- SCN1 (Fig. 3a): QoL decreases by 2% annually after age 50
  - General health decline due to aging.
- SCN2 (Fig. 3b): QoL decreases by 1% annually after age 50
  - Gradual health decline due to aging.
- SCN3 (Fig. 3c): QoL decreases by 1% annually after age 30, remains stable between ages 50 and 70, and decreases by 1% annually after age 70
  - Health deterioration starting in early adulthood due to poor lifestyle habits or chronic illnesses, such as smoking.
- SCN4 (Fig. 3d): QoL sharply declines at age 60 and decreases by 1% annually thereafter
  - Sudden health decline due to accidents or life-threatening diseases.

In this study, SCN denotes Scenario. The SCN1 setting is based on previous studies^51,54^. The other scenarios are adjusted to match the total area of SCN1 by altering the age at which QoL reaches 0. This approach ensures that the total effect of life extension remains constant across all scenarios. Here, we assume that the QoL declines considered in the four scenarios are primarily attributable to health-related factors.

### Method for policy evaluation using QALY

In this study, we numerically compare healthcare policy evaluations using the QALY proposed in this research with those using conventional QALY, focusing on Japan as the target region. The equation representing cost reduction as a policy evaluation is as follows:

4 $$\begin{aligned} \text {Cost Reduction} = \sum _{a}N_{a}\left( QALY_{base} - \sum _{m}\beta _{m}QALY_{m}(a)\right) \end{aligned}$$

Here, $N_a$ and $\beta _{m}$ represent the population at age *a* in the target region and scenario proportion, respectively. Eq. (4) allows us to calculate the difference between the evaluation where QALY is constant regardless of age ($QALY_{base}$) and the evaluation that considers scenarios (and their occurrence rates) and age, as proposed in this study. By multiplying this difference by the population at each age, we can measure the effect of overall social cost reduction, such as healthcare expenses in Japan. This comparison allows us to discuss the role of the current QALY (or CBA) as well as the potential contribution of the QALY proposed in this study.

### Data description and compliance

We consider Japan as the target region for our numerical analysis and use socioeconomic data on income, consumption, survival rates, and population to calculate the VSL and QALY^61–64^. Additionally, in CBA in the medical field in Japan, a value of 5 million yen per QALY is commonly used. Therefore, we assume $QALY_{base}$ to be 5 million yen^30,65^. This study adheres to the Consolidated Health Economic Evaluation Reporting Standards 2022 (CHEERS 2022) guidelines^66^.

## Supplementary Information

Supplementary Information.

## Data Availability

The datasets generated and/or analyzed during the current study are publicly available from the Statistics Bureau of Japan (SBJ) and the Ministry of Health, Labour and Welfare of Japan (MHLW) repository, https://www.stat.go.jp/english/data/kakei/156index.html, https://www.mhlw.go.jp/english/database/db-l/wage-structure.html, https://www.stat.go.jp/english/data/jinsui/2.html. The source code used for the analysis is publicly available at the following repository: https://github.com/kazz2817/QALY-code. For further inquiries or to request data, please contact the corresponding author at kazu-ito@rs.tus.ac.jp.
