# Supplementary material for: Deriving monetary value of quality-adjusted life years through life extension from the value of a statistical life
Source: Sci Rep. 2025 Dec 1;16:341. doi: 10.1038/s41598-025-29794-6 (PMC12770314; doi:10.1038/s41598-025-29794-6)
Supplement: Supplementary file 1 — Supplementary Information. [file 41598_2025_29794_MOESM1_ESM.pdf]

Deriving Monetary Value of Quality-Adjusted Life Years through Life Extension from the Value of a Statistical Life

Yusuke Tanizawa, Kazuya Ito, Ryuta Takashima

Supplementary Table

Supplementary Table S1. The value of QALY for representative ages under different interest rates (million JPY)

		SCN1			SCN2			SCN3			SCN4		
Interest rate [%]		1	2	3	1	2	3	1	2	3	1	2	3
Age	20	3.54	1.97	1.12	3.11	1.80	1.06	3.74	2.08	1.18	3.59	2.00	1.14
	50	4.61	3.38	2.51	4.01	3.05	2.35	5.99	4.40	3.27	4.68	3.43	2.56
	80	9.43	8.53	7.73	4.94	4.63	4.35	7.78	7.09	6.48	9.11	8.27	7.51

Supplementary Table S2. The value of QALY for representative ages under logarithmic utility function (million JPY)

		SCN1	SCN2	SCN3	SCN4
Age	20	3.58	3.13	3.81	3.64
	50	4.67	4.05	6.11	4.75
	80	9.85	5.22	8.21	9.56

Supplementary Table S3. The value of QALY for representative ages under constant relative risk aversion (CRRA) utility function (million JPY)

		SCN1	SCN2	SCN3	SCN4
Age	20	3.56	3.12	3.78	3.62
	50	4.64	4.03	6.07	4.73
	80	9.69	5.12	8.05	9.39

Supplementary Table S4. The value of QALY for representative ages under hyperbolic discounting (million JPY)

		SCN1	SCN2	SCN3	SCN4
Age	20	3.43	2.98	3.62	3.48
	50	4.33	3.77	5.63	4.40
	80	9.09	4.81	7.52	8.79

Supplementary Note

A key related study is Hammitt (2023), which employs time-varying hazard rate reductions and health-related QoL weights to derive the age-specific value per statistical life (VSL), value per statistical life year (VSLY), and value per quality-adjusted life year (VQALY). Although the mathematical structures of that framework and ours differ, Hammitt's VQALY is essentially equivalent to the value of QALY in our study and shares the same interpretation and implications. By using Eqs. (4), (5), and (9) – (11) in Hammitt (2023), the relationship among VQALY, VSLY, and the present value of one QALY (QALE) can be expressed as follows (See Hammitt (2023) for detailed definitions of each variable):

$$VQALY(x) = \frac{VSLY(x)LE(x)}{QALE(x)} \tag{S.1}$$

where $LE(x)/QALE(x)$ is calculated as follows:

$$\frac{LE(x)}{QALE(x)} = \frac{\frac{1}{s(x)\delta(x)}\int_x^\infty s(t)\delta(t)dt}{\frac{1}{s(x)\delta(x)}\int_x^\infty q(t)s(t)\delta(t)dt} = \frac{1}{\overline{q}(x)} \tag{S.2}$$

where $\overline{q}(x)$ is the weighted average health-related QoL at a given age x. Substituting this into the relationship between VQALY, VSLY, and QALE, VQALY is expressed as:

$$VQALY(x) = \frac{VSLY(x)}{\overline{q}(x)} \tag{S.3}$$

In this sense, $VSLY(x)$ directly corresponds to our $LEV_m(a)$, and $\overline{q}(x)$ to our $H_m(a)$, carrying the same substantive interpretation. Accordingly, although Hammitt's (2023) and our model framework employ different methodologies, they essentially provide equivalent representations of the value of QALY.
